# Elevational Patterns in Archaeal Diversity on Mt. Fuji

**DOI:** 10.1371/journal.pone.0044494

**Published:** 2012-09-06

**Authors:** Dharmesh Singh, Koichi Takahashi, Jonathan M. Adams

**Affiliations:** 1 Department of Biological Sciences, College of Natural Sciences, Seoul National University, Seoul, South Korea; 2 Department of Biology, Faculty of Science, Shinshu University, Matsumoto, Japan; 3 Institute of Mountain Science, Shinshu University, Matsumoto, Japan; Argonne National Laboratory, United States of America

## Abstract

Little is known of how archaeal diversity and community ecology behaves along elevational gradients. We chose to study Mount Fuji of Japan as a geologically and topographically uniform mountain system, with a wide range of elevational zones. PCR-amplified soil DNA for the archaeal 16 S rRNA gene was pyrosequenced and taxonomically classified against EzTaxon-e archaeal database. At a bootstrap cut-off of 80%, most of the archaeal sequences were classified into phylum Thaumarchaeota (96%) and Euryarchaeota (3.9%), with no sequences classified into other phyla. Archaeal OTU richness and diversity on Fuji showed a pronounced ‘peak’ in the mid-elevations, around 1500 masl, within the boreal forest zone, compared to the temperate forest zone below and the alpine fell-field and desert zones above. Diversity decreased towards higher elevations followed by a subtle increase at the summit, mainly due to an increase in the relative abundance of the group I.1b of Thaumarchaeota. Archaeal diversity showed a strong positive correlation with soil NH_4_^+^, K and NO_3_^−^_._ Archaeal diversity does not parallel plant diversity, although it does roughly parallel bacterial diversity. Ecological hypotheses to explain the mid diversity bulge on Fuji include intermediate disturbance effects, and the result of mid elevations combining a mosaic of upper and lower slope environments. Our findings show clearly that archaeal soil communities are highly responsive to soil environmental gradients, in terms of both their diversity and community composition. Distinct communities of archaea specific to each elevational zone suggest that many archaea may be quite finely niche-adapted within the range of soil environments. A further interesting finding is the presence of a mesophilic component of archaea at high altitudes on a mountain that is not volcanically active. This emphasizes the importance of microclimate – in this case solar heating of the black volcanic ash surface – for the ecology of soil archaea.

## Introduction

In the three decades since the discovery of archaea [1] and the two decades since its formal recognition as a new domain of life [2], our understanding of archaeal biology, ecology and evolution has expanded considerably. While archaea were at first regarded as life forms confined to the extremophilic environments, based on the characterization of cultivated specimens found in such environments [2], [3], they have now been found in a wide variety of habitats both exotic and mundane, including hydrothermal vents, [4], marine waters, [5], [6], marine sediments [7]–[9], freshwater sediments, [10]–[13], soil [14]–[18], subsurface goldmine [19], the hindgut of termites [20] and the casts of earthworms [21], thereby breaking the ‘extremophilic stereotype’ [22]. The detection of archaea by molecular screening of 16S rRNA gene, without cultivation, has significantly improved knowledge of this group and has added many major new groups, with the latest entrant being Thaumarchaeota [23]. Thaumarchaeota - initially classified as mesophilic crenarchaeota - are amongst the most abundant archaea on Earth, found in a wide variety of ecosystems including soils, marine and fresh waters as well as in moderately extremophilic environments.

However, even though over a hundred archaeal genome sequences are publicly available [24] and our knowledge of the archaea has increased substantially but still our understanding of its diversity and community ecology is limited. Several broad scale surveys have already provided scattered phylogeographical clues to the diversity patterns and ecology of soil archaea [25], [26]. For instance, Auguet et al. [25] provided valuable insight into broad-scale ecological patterns exhibited by the archaeal domain in general, using around 2000 archaeal 16S rRNA environmental sequences available online from a large set of environments and utilizing this information to extract general macroecological patterns found among archaeal communities along global environmental gradients. Similarly, Bates et al. [26] sought the environmental factors which regulate the diversity and abundance of archaeal communities in soil with 146 samples from the Americas and Antarctica. These two major studies and other recent investigations suggest that archaeal communities can be influenced by salinity and pH, [25], [27], elevation, [28], climate and vegetation cover [29] or C/N ratio [26]. To the best of our knowledge, only the study by Zhang et al. [28] has looked at archaeal diversity along an elevational gradient. This study showed that the abundance of ammonia oxidizing archaea (AOA) was negatively correlated with altitude.

In studies of elevational gradients in macroorganisms like vertebrates, larger invertebrates and higher plants, two trends have often been found: a monotonous decline or a humpback trend in species richness with increasing elevation [30]–[32]. Tree and bird species diversity typically declines with elevation, or a bulge is seen only at lower elevations [31], [33], [34], whereas in the case of mammals or amphibians a mid-elevation peak is more often observed [32], [34]–[36]. In the case of prokaryotes, bacterial diversity trends have been studied several times along elevational gradients, yielding altogether different results: either no trend [37], a monotonous decline [38] or a humpbacked trend [39] with increasing elevation. So far there have been no studies of the full taxonomic diversity of soil archaea with respect to elevation. Mountain systems represent unique opportunities to study soil characteristics that influence archaeal community composition and diversity, where variation of other environmental factors (e.g., geology or climate) that could confound results is minimized.

In this study, we sampled archaea from soil on an elevational gradient on Mt. Fuji and asked the following exploratory questions: (i) what are the dominant archaeal phyla in Mt. Fuji soil, and how does their relative abundance vary with elevation? (ii) How does the overall archaeal diversity vary along this elevational gradient of Fuji? (iii) What environmental factors predict soil archaeal community structure? Although it is difficult to formulate precise hypothesis, due to lack of studies on this topic, we nevertheless expected that: a) Thaumarchaeota would be equally dominant at all elevations, b) archaeal diversity would be related to one or more identifiable soil parameters, and c) archaeal diversity would decrease with altitude as was found for AOA on Mt. Everest.

## Results

### Community Composition

**Figure 1:**
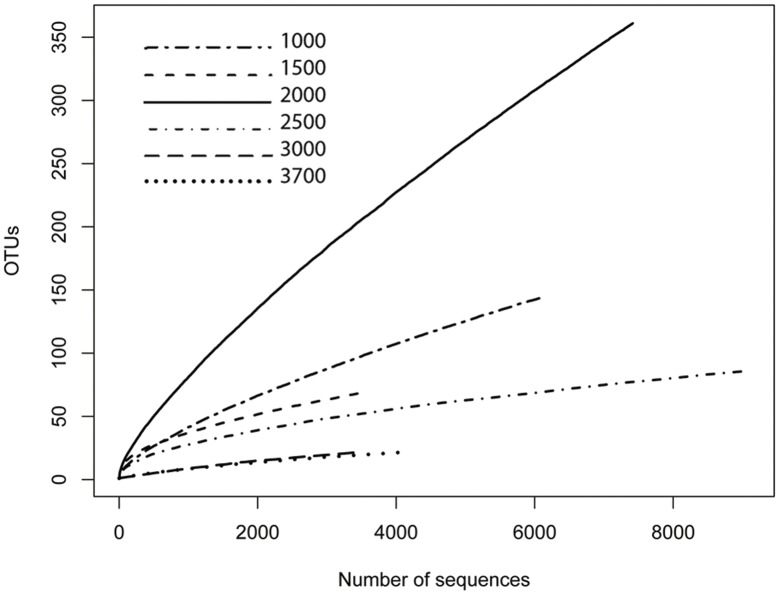
Rarefaction analysis of one example from each elevational set of samples calculated for the 0.3 OTU definition (defined at ≥97% sequence similarity level) based on pairwise distance. Each line type denotes the elevation (meters above sea level) from which the samples were collected.

**Figure 2:**
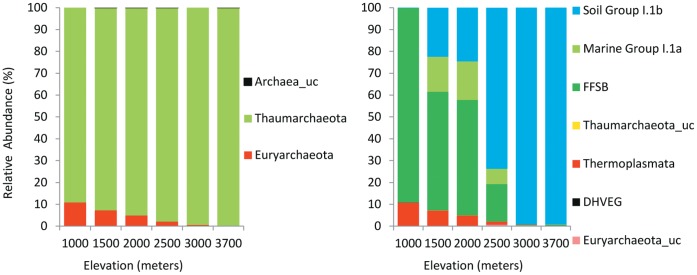
Relative average abundances of archaeal taxa at different elevational sampling points at the phylum level (left) and at the sub-phylum level (right). See Table S2 for additional taxonomic descriptions. (Abbreviations: FFSB- Finnish Forest Soil archaea type B; _uc-unclassified).

Based on the results of Kan et al. [40] and our supplementary analysis (see Document S1), we decided to use EzTaxon-e to assign the taxonomy to our recovered sequences, as a better database for this task. A total of 89672 quality archaeal sequences (with an average length of 444 bp) were obtained from the 30 samples, with an average of 2989 sequences per soil sample and with coverage ranging from 398 to 8488 reads per sample. Even with this level of coverage, the lack of asymptotes in the rarefaction curves (Fig. 1) suggests that much archaeal diversity remains un-sampled. Out of a total 89016 sequences that remained after the trimming, aligning and screening processes, around 99.9% sequences could be classified up to phylum level with a total 1478 phylotypes (defined at ≥97% sequence similarity level) (see Table S1). Thaumarchaeota emerged as the most abundant archaeal phylum on Mt. Fuji with 85840sequences, (96.4%) of the total across all elevations, although it was more abundant at higher elevations. Euryarchaeota was the only other phylum present on Mt. Fuji (3515sequences, 3.9%) with a trend opposite to that found for the Thaumarchaeota: a higher relative abundance at lower elevations which progressively decreased to an almost negligible presence at the summit (Fig. 2).

**Figure 3:**
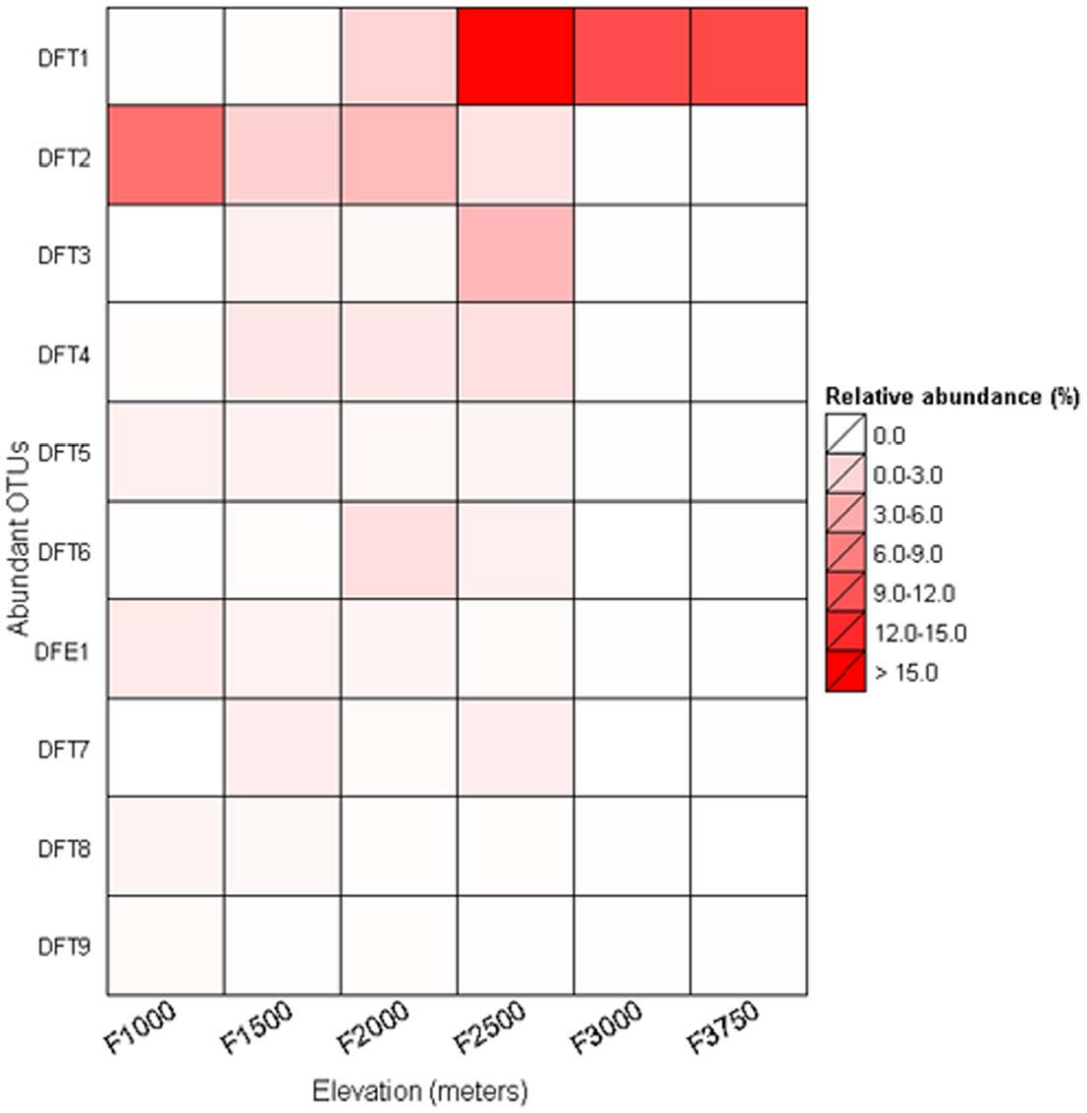
Heat map showing the percent relative abundance of the 10 most abundant phylotypes at different elevational sampling points with a color legend and scale provided. DFT here abbreviates for Dominant Fuji Thaumarchaeota and DFE for Dominant Fuji Euryarchaeota. The number written against them denotes their abundance e.g., DFT1 stands for the most abundant thaumarchaeotal phylotype present on Mt. Fuji.

The most abundant single phylotype across the entire sample was classified under the order Nitrososphaerales (soil cluster I.1b, Thaumarchaeota) represented by a total of 41,433 sequences accounting for approximately 46.5% of total classifiable sequences (see Table S1). This particular OTU increased in relative abundance with increasing elevation. It was almost absent at the lowest elevation, but reached around 20% to 70% at the mid elevation sites, and then dominated at high elevations where it represented nearly all of the sequences recovered at 3000 and 3750 masl (see Table S1). Of the 10 most abundant OTUs, (94.5% of the total sequences; Fig. 3) only 3 belonged to Euryarchaeota, all of them within the class Thermoplasmata. Most of the abundant thaumarchaeotal and euryarchaeotal OTUs were present in their greatest numbers at lower/mid altitudes except for the most abundant phylotype DFT1(Dominant Fuji Thaumarchaeota 1) which was more abundant at higher altitudes (Fig. 3).

### Archaeal Diversity Along the Elevational Gradient

**Figure 4:**
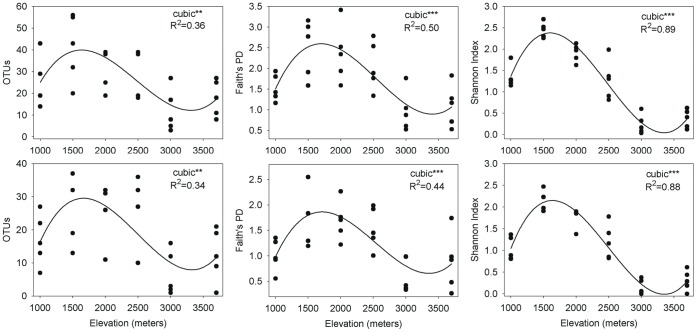
Relationship between elevation and phylotype richness (left), phylogenetic diversity (middle), phylotype diversity (right) in the whole community (first row) and Thaumarchaeota (second row). We tested three models (linear, quadratic, and cubic) to describe the relationships and model selection was carried out based on adjusted R^2^ and RMSE (root mean square error; value not shown). Significance level is shown with ***P<0.001; **P<0.01; and P<0.05.

The archaeal communities rarified to the same level of subsampling (309 reads per sample), showed significant differences in diversity and richness in relation to elevation (Fig. 4, see Table S2). There was a “peak” in diversity/richness in the lower mid-elevations at around 1,500 masl with a curve showing the best fit based on adjusted R^2^ and residual standard mean error. Maximum richness with approximately 79% of OTUs was observed at 1500 masl whereas minimum richness was observed at 3000 masl. Richness at the summit was lower than that observed at the lowest elevation 1000 masl, with only 92.4% as many OTU’s present at the summit.

Among all the site characteristics examined, elevation was most significantly correlated (P<0.05) with both OTU richness (R^2^ = 0.36) and diversity (Shannon index, R^2^ = 0.89; Faith’s PD, R^2^ = 0.50) (Fig. 4). The same analysis at a subsampling level of 1000 reads with only 22 samples showed results with similar values (results not shown). When we further examined the most dominant phylum Thaumarchaeota, richness and the phylogenetic structure once again correlated most strongly with elevation as supported by the adjusted R^2^ values and corrected Bonferroni P values (Fig. 4). Among all edaphic variables, extractable ammonium, nitrate and potassium ion concentration also showed a positive correlation with both richness and diversity (Table 1). No relationship was found between C/N ratio and richness but C/N ratio was correlated to both diversity measures; soil pH was only correlated with Shannon diversity index (Table 1).

**Table 1:** Relationship between soil parameters and phylotype richness, phylogenetic diversity and phylotype diversity for the whole community and Thaumarchaeota.

Variables	OTUs	Faith’s PD	Shannon Index
**Whole community**			
CN ratio	–	0.13(l*)	0.24(l**)
pH	–	–	0.35(q**)
Total Carbon	0.18(l*)	0.17(l*)	0.58(q***)
Ammonia	0.28(l**)	0.21(l**)	0.56(q***)
Nitrate	0.27(l**)	0.27(l**)	0.34(q**)
Phosphorus	–	0.21(q*)	0.44(q***)
Elevation	0.36(c**)	0.50(c***)	0.89(c***)
Potassium	0.29(l**)	0.35(q**)	0.70(q***)
**Thaumarchaeota**			
CN ratio	–	0.13(l*)	0.21(l**)
pH	–	–	0.23(q*)
Total Carbon	0.15(q*)	–	0.46(q***)
Ammonia	0.15(l*)	0.17(l**)	0.52(q***)
Nitrate	0.12(l*)	0.29(l**)	0.34(q**)
Phosphorus	–	0.18(q*)	0.40(q***)
Elevation	0.34(c**)	0.44(c***)	0.88(c***)
Potassium	0.22(l**)	0.30(q**)	0.64(q***)

We tested three models (linear-l, quadratic-q, and cubic-c) to describe the relatioships; model selection was carried out based on adjusted R^2^ and RMSE (root mean square error; value not shown). Significance level was shown with***P<0.001;

** P<0.01; and P<0.05; only relationships which were significant are shown in table.

**Figure 5:**
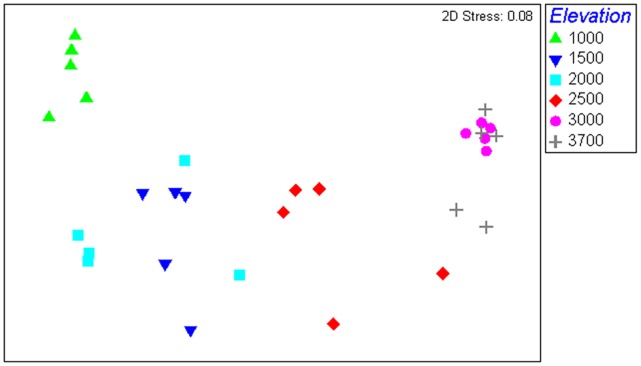
NMDS analysis results with a Bray Curtis similarity matrix comparing all 30 samples from 6 different elevational points from Mt. Fuji.

Elevation acted as a strong structuring factor of the archaeal assemblages showing that samples belonging to different elevational zones harbored distinct communities, based on the Bray-Curtis index (Fig. 5).

We assessed the relative importance of environmental variables in explaining their contributions to the correlation using MRM. Only 5 out of the 9 environmental variables were used for this analysis (see Methods and Fig. S1). For the whole community (both UniFrac and Bray-Curtis matrices), elevation alone was able to predict more than 38% of the total variability (Table 2). Apart from elevation, potassium ion concentration was also able to explain a smaller portion (around 18%) of the variation.

**Table 2:** Results of the multiple regression on matrices analysis for the whole community.

Environmental Variables	Whole community	
	Bray-Curtis (R^2^ = 0.63b)	UniFrac (R^2^ = 0.38b)
pH	–	–
Sqr (Elevation)	−14.8***	0.032***
Ln (P)	–	−0.012*
Sqr (K)	−4.5**	0.013*
NO_3_^−^	–	–

The variation (R^2^; both values are significant at P≤0.0001) of community distance that is explained by the remaining variables and the partial regression coefficients (b) of the final model is reported. Partial regression coefficients are reported for only significant values (*P≤0.0100, **P≤0.0010, and ***P≤0.0001).

## Discussion

### Diversity and Elevation

Archaeal diversity on Fuji, as with bacteria [39], shows a mid-elevation “peak”, although at a lower elevation of 1500 masl than in the case with bacteria. This ‘humpback’ trend contrasts with the trend in vascular plant species richness on Mt. Fuji, where both tree and herbaceous plant richness steadily declines with increasing elevation [41]. A greater variety of plant species might be expected to provide more diverse environments for soil microbes, but since archaea do not tend to be involved in litter decomposition in contrast to bacteria or fungi [42], [43]; it is not surprising that they show an independent trend. The only comparable study of archaea with elevation has been that by Zhang et al. [28] on Mount Everest (12 soils at altitudes of 4000–6500 masl). That study concentrated only on ammonia oxidizing microbes, showing a significantly negative correlation with altitude, with a maximum in abundance at the lowest altitudes. Our results differ from those of Zhang et al. [28]. However, the taxonomic and environmental sampling range studied here is very different; they also examined only a limited number of sites over each elevation range, with almost no replicates except three at the lowest sampling site at 4000 masl.

A humpback trend in diversity with elevation is quite commonly found in groups of animals and plants in mountains around the world [31]–[34], most often towards the lower altitudes. However, a monotonous decline in diversity is also very common for a wide range of groups [31]–[34]. A range of hypotheses have been put forward for such trends [31], including intermediate disturbance intensity, a ‘mid domain effect’, and the effects of combining the communities of two relatively distinct environments (from upper and lower slopes) in the intermediate elevations [39].

As in our previous study of bacterial diversity on Fuji [39], the mid elevation ‘bulge’ in diversity might be explicable in terms of several different factors/processes [34]. It is possible that a more physically stable soil environment in the lowermost forest zone of Fuji allows out-competition between archaeal species with overlapping niches, reducing overall diversity. The very unstable upper slopes of Fuji, with bare alpine ash/clinker fields subject to frost heave, landslips and avalanches, may provide the opposite extreme of an environment in which few species can maintain viable populations (or in which few niches are viable due to frequent population reductions) – hence the lower diversity of the upper elevations [34].

Another possibility is that the mid-altitudes of Fuji in effect combine a small-scale mosaic of two environments: the upper slope unstable environment of the ash/clinker fields, and the lower slope stable forest soil environment. This is a variant of the hypothesis of Lomolino [31]. The combination of two distinct environments, and their associated archaeal communities, on a micro-scale in the mid-altitudes of Fuji could increase diversity by adding together two sets of species. This demands further investigation through fieldwork observations, experiments, and microcosm studies.

Potentially very important however, are the observations of relationships between diversity and soil parameters. These may hint at other mechanisms that control diversity at the level of resource availability, perhaps mediated by competition or by the availability of extra niches. Potassium, ammonium and nitrate concentrations are all significantly correlated with diversity, although none as strongly as elevation itself. Since potassium and ammonium concentrations co-vary, their relative importance is difficult to discern, and they might all perhaps be correlated with some unknown factor (also related to elevation, such as disturbance) which could be in fact the most important in controlling the diversity trend. Again, further studies are necessary to elucidate this.

### Community Composition and Elevation

Elevation was significantly correlated with both the composition of the whole community and the relative abundance of subgroups within the major phylum Thaumarchaeota (Table 1 & 2; Fig. 4 & 5). All of the statistical analyses emphasize the overwhelming predictive power of elevation as a principal driving force in the soil archaeal community on Mt. Fuji. The explanation to this strong correlation with elevation may be the strong co-variation of different soil edaphic variables with elevation (VARCLUS results, Fig. S1). Among these elevation-dependent variables, extractable potassium ion concentration also has a particularly strong influence (Table 2) on the community structure and phylogeny. Interestingly, the archaeal diversity bulge at 1500 masl coincides with the maximum values of most of the soil variables we studied, except pH and total carbon (soil and site characteristics previously described in Singh et al [39]). Earlier studies on soil archaeal communities from elsewhere found a negative correlation between soil archaeal abundance/diversity and pH [27], [44]–[46] but in this study pH was generally not found to be significant. However, pH range was quite narrow on Mt. Fuji, with a general pH gradient from lower elevations to higher ones (4.8 to 6.4).

Two other studies that have concentrated on broad scale differences in soil archaeal communities concluded that salinity [25] and C/N ratio [26]) is the principal driving force behind archaeal taxonomic distribution at global scales. Auguet et al. [25] collected c.2000 sequences of the archaeal 16S rRNA gene from 67 globally distributed studies with samples ranging from hydrothermal vents to chemical reactors including water and sediments samples from freshwater and marine environments, and soil samples. Their study focused on how ecology relates to the community structure and therefore it may not be directly comparable with the soil gradient we explore here. Bates et al. [26] on the other hand, collected 146 soil samples from North and South America and Antarctica resulting in a total of 2500 sequences corresponding to archaea. They primarily examined the influence of environmental factors on archaeal abundance relative to that of soil bacteria. Although both studies took a global perspective, the total number of sequences taken in for consideration at 2000 and 2500 reads, were rather few. In contrast, our study recovered around 80,000 archaeal sequences from 30 samples examined here, which allowed for a more comprehensive assessment of archaeal diversity in these soils. Also, it is interesting to note that despite the greater number of sequences, the soils in our study were still dominated by very few archaeal taxa as has been observed before [16], [25], [26], [47].

At lower elevations, the dominance of FFSB (I.1c gp) of Thaumarchaeota cannot be ignored. It has been seen in many previous studies that archaeal communities in acidic forests are dominated by the FFSB group [16], [46]. Soil pH is a major determinant of the abundance of FFSB group with lower abundance at neutral/higher pH values and vice versa. Lehtovirta et al. [46] sampled across pH manipulated plots in the range of 4.5 to 7.5 (maintained at 0.5pH unit intervals) to study whether soil pH is a major driver of FFSB group and found that FFSB could be detected only in soils at pH 4.5 to 6.0 with highest abundance at the lowest pH accompanied with a steady decline as pH increased. This may explain the dominance of FFSB at lower elevations, where the pH is comparatively lower as compared to higher elevations [39].

We found an overall shift away from Euryarchaeota towards Thaumarchaeota abundance (relative abundance, Fig. 2) with increasing elevation. The Euryarchaeota assemblage present on Mt. Fuji was almost entirely composed of sequences that could be classified into class Thermoplasmata. Thermoplasmata is a large class consisting of thermoacidphiles (pH optima 0.7 to 3 and optimum temperatures above 50°C – based upon cultured specimens) which are aerobic or microaerophilic heterotrophs [48]. Increase in Thaumarchaeota towards upper elevations was mostly due to increasing prevalence of the thaumarchaeotal soil cluster I.1b (see Fig. 2, right). Looking at a finer taxonomical scale, this increase was largely due to a single but most abundant (53.7%) OTU cluster DFT1 (designated ‘dominant Fuji thaumarchaeota 1′) classified under thaumarchaeotal soil group I.1b. Our results are in accordance with previous soil studies, where the majority of the archaeal phylotypes were contained within the same lineage of Thaumarchaeota (i.e., soil I.1b clade, earlier classified under crenarchaeota) [13], [26], [49].

**Figure 6:**
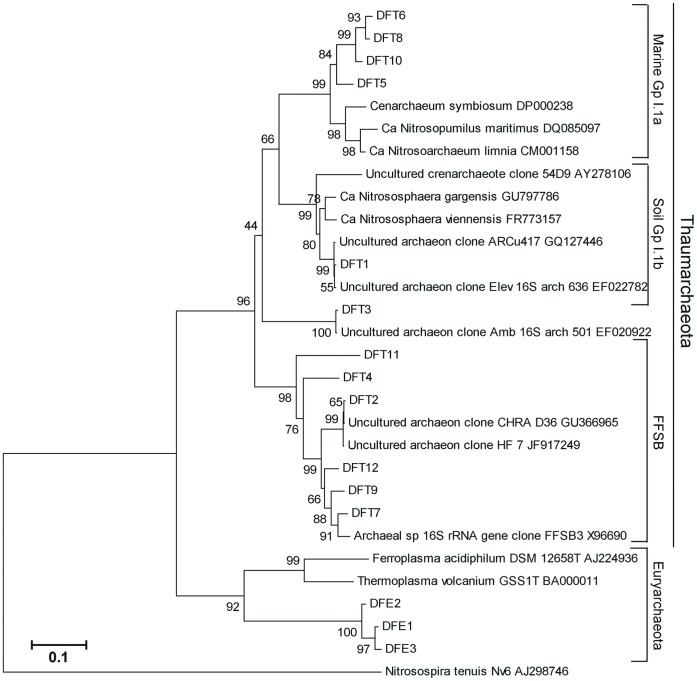
Neighbor joining tree based on the alignment of 16S rRNA gene sequences (∼400 bp long) showing the relationship between archaeal phylotypes (DFT: Dominant Fuji Thaumarchaeota and DFE: Dominant Fuji Euryarchaeota) recovered from Mt. Fuji by pyrosequencing. The dominant soil thaumarchaeote DFT1 is indicated along representative archaeal isolates and clone 54d9. The tree is rooted with an AOB from bacterial phylum Proteobacteria.

Interestingly, while our samples at 2500 masl and above were overwhelmingly dominated by sequences belonging to soil group I.1b (see Fig. 2 and Table S1), these were present at lower elevations in only minimal numbers. Bates et al. [26] had earlier suggested that soil group I.1b could be an AOA as it formed a tight clade with the uncultured soil clone ‘54d9’, a large genomic fragment obtained from a soil fosmid library that included the entire 16S/23S rRNA gene, [49], [50] as this clone was shown to contain genes encoding ammonia monooxygenase (Amo)-related proteins [50]. A phylogenetic tree (Fig. 6) incorporating the 15 most abundant phylotypes including ‘DFT1’ on Mt. Fuji with sequences for uncultured clone 54d9 and other fellow AOA from the soil cluster I.1b like *Nitrososphaera gargensis* (GU797786), *N. viennensis* (FR773157), and *Cenarchaeum symbiosum* (DP000238) revealed DFT1 within a tight clade with the other AOA and the uncultured clone 54d9. This suggests that DFT1 could be a possible member of the AOA clade and that the substantial increase in the soil cluster I.1b at higher elevations on Mt. Fuji could be due to an increase in abundance of AOA.

Dominance of *Nitrososphaera*-like AOA at higher elevations where the mean annual temperature (MAT) is much lower than 5°C, contrasts with findings of culture studies of other AOA belonging to group I.1b like *N. gargensis* (optimum temperature 46°C, [51]), *N. viennensis* (optimum temperature 35°C, [52]) and strain JG1 (optimum temperature 35–40°C, [53]). This point could be explained by the fact that even though the atmospheric temperature is much lower than required for the cultivated gp I.1b AOA, the temperature of a soil surface exposed to strong solar radiation is much warmer than the atmospheric temperature [54]. According to this report by Masuzawa [54], the maximum soil temperatures exceeded 50°C for several days in July and August, and were regularly above 40°C on Mt. Fuji on partly vegetated black volcanic soil at the timberline, located at 2500 masl. We sampled at the same time of year – late July 2010. Keeping such observations in mind, and given the black-colored volcanic ash/scoria present on most of the upper half of Mt. Fuji, almost free of vegetation, we may expect soil temperatures as high or even higher than Masuzawa reported, as soil temperatures tend to remain closer to mean air temperatures under trees than under treeless vegetation or no vegetation [55]–[57]. Direct solar heating of the open volcanic ash soils may thus provide suitable temperatures for *Nitrososphaera*-like AOA at higher elevations.

In earlier reports, substrate uptake assays have shown that the affinity of AOA for ammonia was much higher than ammonia oxidizing bacteria (AOB) [53], [58]–[61] which indicates that AOA may be physiologically adapted to ammonia oxidation in environments with low concentrations of ammonia. Clearly, it is likely that a variety of environmental variables determine the relative contribution of AOA to soil nitrification, although it is generally agreed that AOA have a competitive advantage at low concentrations of ammonia/ammonium [62], [63]. Strain JG1(pH range:6–8, optimum temperature:35–40°C, ammonia tolerance up to 20 mM, [53]), Ca. *N. viennensis* (optimum pH:7.5, optimum temperature:35°C, ammonia tolerance up to 15 mM, [52]) and Ca. *N. gargensis* (optimum pH:7.4, optimum temperature:46°C, ammonia tolerance up to 3.08 mM, [51]) are the only described archaea affiliated with thaumarchaeotal gp I.1b suggesting that the archaea affiliated to this group are generally mesophiles (20°C–50°C) which prefer near neutral pH conditions and have been found to tolerate ammonia/ammonium up to a concentration of 20 mM. This might explain why there is a surge in abundance of the soil group I.1b (OTU DFT1) at higher altitudes above 2000 masl: soil ammonium concentrations rapidly decrease above 2000 masl to reach concentrations of around 20 mM, combined with high soil temperatures (often 40–50°C) and suitable pH (6.2 at higher altitudes) on Mt. Fuji. It is also important to note that group I.1b AOA are consistently predominant over group I.1a AOA [25], [49], [64] and AOB [44], [47], [65], [66] in terrestrial environments and such high occurrence of DFT1 here on Mt. Fuji higher altitudes (where the environmental conditions are quite optimum for a group I.1b AOA) could be a just a norm. Although our results and this explanation above are not definitive proof that archaeal community composition in higher altitude soils is dominated by AOA, they suggest that DFT1 could be an AOA.

In conclusion, our results have revealed a humpback diversity pattern for archaea along an elevational gradient. The most important findings of this study are: 1) Soil archaeal communities and their diversity are strongly responsive to environmental gradients on the scale of a single mountain. The humpback trend may be a consequence of the various environmental parameters which co-vary with elevation on Mt. Fuji, including temperature, vegetation type or soil nutrients such as ammonium and potassium. The finding of relatively discrete communities of archaea specific to each elevational zone suggests that many archaea may be quite finely niche-adapted within the range of soil environments. 2) A further interesting finding is the presence of a thermophilic component of archaea at the soil surface at high altitudes on a mountain that is not volcanically active. This emphasizes the importance of microclimate – in this case solar heating of the black volcanic ash surface – for the ecology of soil archaea.

This study also revealed an elevational gradient in relative abundance of Thaumarchaeota vs. Euryarchaeota, and amongst the various classes of the Thaumarchaeota. Groups of Thaumarchaeota which are likely to contain ammonium oxidizers become relatively more abundant towards the summit of Fuji. Further work is needed to understand the underlying causes of these patterns, including both additional observational studies along gradients, and experiments involving manipulation of soil conditions. Soil manipulation experiments to better understand the controls on archaeal community structure and diversity should focus on: 1) artificial opening and disturbance of the vegetation below the tree line to simulate the hypothesized role of disturbance in producing the observed patterns; 2) transplantation of small quantities of soil between various elevations to understand the role of temperature in controlling the characteristic microbial communities found in each elevational zone, and 3) shading experiments to understand the importance of soil direct heating by the sun in producing the thermophilic community found on the upper parts of Fuji.

## Materials and Methods

### Sampling, Site Description, Soil Characterization and DNA Extraction

Sampling was carried out along the elevational gradient on Mt. Fuji (35^o^21’28.8″N 138^o^43’51.6″E) starting from the base of the mountain at 1000 masl, to the summit area at around 3760 masl. We sampled along a transect on the north face of the mountain (Subaru Trail) at 6 sampling elevational zones. At each sampling level, we took 5 samples separated by approximately 500 m horizontally on the same elevational contour, as previously described by Singh et al. [39], resulting in a total of 30 samples. At each elevational sampling level, we sampled soil from five 10 m×10 m squares. In each square, approximately 100 g of the top 5 cm of B-horizon soil (defined as any mineral particle present) was taken from each corner and the center point of the square; the five samples were well mixed into one bag to provide a composited sample. Soil samples were then sieved (3 mm) and stored at −80°C within 24 hrs. The northern face of Fuji has not been affected by any eruptions in the last 10 000 years, unlike lower parts of the south face which experienced a flank eruption several centuries ago, yielding ash which fell down slope and to the south-east [Mt. Fuji Volcano Disaster Management Conference (2002) available from: http://www.bousai.go.jp/fujisan-kyougikai/. Japanese government report (in Japanese)]. All soil samples were collected within a single day in the last week of July 2010, historically the warmest week of the year and after all snow had melted away from the mountain. Details of the site/soil characteristics, climate/vegetation zones, geological background of Mt. Fuji and the procedure utilized for the soil analysis and DNA extraction have been previously described in detail by Singh et al. [39]. Since all the soils are below neutral pH, it is unlikely that much of the nitrogen would be present as ammonia, hence we did not analyze for ammonia, only ammonium. DNA isolated was stored at −80°C and was utilized as such for the PCR amplification.

### PCR Amplification and Pyrosequencing

PCR amplification used bar-coded primers targeting the V1 to V3 region of the 16S rRNA gene, with PCR conditions and primers as previously described by Hur et al. [67]. Briefly, PCR reactions were performed in 50 µl reactions, each containing 1 µl (20 nm) of both primers, 5 µl (PCR reaction buffer with MgCl_2_, 10X), 1 µl (dNTP mix), 0.25 µl (Taq DNA Polymerase, 5 U/µl) (Roche Diagnostics GmbH, Mannheim, Germany) and 1 µl of DNA as template. We used the following PCR conditions: initial denaturation 94°C, 5 min, followed by 10 cycles (denaturation, 94°C, 30 s; annealing, 60°C to 55°C with a touch-down program for 45 s; elongation, 72°C, 90 s) tailed by an additional 20 cycles (denaturation, 94°C, 30 s; annealing, 55°C, 45 s; elongation, 72°C, 90 s). Pooled reactions were purified using the QIAquick PCR purification kit (Qiagen) and quantified using PicoGreen (Invitrogen) spectrofluorometrically (TBS 380, Turner Biosystems, Inc. Sunnyvale, CA, USA). 50 ng of PCR product for each sample was combined in a single tube and sent to Chunlab Inc. (Seoul, Korea) for pyrosequencing using Roche/454 GS FLX Titanium platform.

### Processing and Pyrosequencing Data and Taxonomic Analysis

The sequence data obtained after pyrosequencing were processed using Mothur [68] except for the step of removing chimeric sequences. To begin with, sequences shorter than 150 nt with homo-polymers longer than 8 nt and all reads containing ambiguous base calls or incorrect primer sequences were removed. Next, the sequences were aligned against the EzTaxon-e database and then trimmed, so that subsequent analyses were constrained to the same portion of the 16 S rRNA gene (V1–V3 region). Putative chimeric sequences were detected and screened using a similarity-based approach, which splits each query sequence into two even length fragments and then assigns each fragment to a taxon using BLAST search against EzTaxon-extended database (http://eztaxon-e.ezbiocloud.net/; [69] followed by removal of the sequences when two fragments differ at the order level or percent identities are greater than 95% for both fragments despite assigned to different taxonomies. The remaining reads were pre-clustered using the pre-cluster command (http://www.mothur.org/wiki/Pre.cluster) to remove erroneous sequences derived from sequencing errors and then clustered using Mothur’s average algorithm. Taxonomic classification of each OTU (clustered at 97% sequence similarity) was obtained by classifying alignments against EzTaxon-e reference archaeal taxonomy and non-redundant nucleotide archaeal databases files using the classify command at 80% Bayesian bootstrap cutoff with number of iterations as 1000. DNA pyrosequences are available under the following GenBank SRA Accession No. SRA050374.1.

### Statistical Processing and Analysis of Results

Operational taxonomic units (OTUs) (at ≥97% similarity) and other diversity units such as Shannon, Faith’s PD etc., and rarefaction values were calculated using the Mothur platform [68] on a subset standardized to 309 reads per sample using the sub.sample command (http://www.mothur.org/wiki/Sub.sample) in Mothur. This subset was used to assess the relationships between OTUs and diversity indices with elevation and other edaphic factors by correlation analysis. Best fitting modeling of correlations were performed in SigmaPlot, using linear, polynomial (quadratic) and power (cubic) law functions. To evaluate if 309 reads per sample are representative of the patterns observed, we repeated the regression analyses using a subsampling size of 1000 reads (available only for 22 samples). OTUs and other diversity metrics were also calculated for the largest phylum Thaumarchaeota and analyzed in the same way as for the whole community.

Community similarity matrices for analysis were built using the Bray–Curtis similarity coefficient [70] and the UniFrac metric [71]. UniFrac is a phylogenetic metric which measures the distance between communities based on the lineages they contain. UniFrac distances were calculated based on a phylogenetic tree of randomly chosen subsets (n = 309reads/subset) of 30 samples. Sequences aligned using Mothur software were used to infer a maximum likelihood (ML) tree using RAxML [72]. RAxML (v.7.2.7) with GTR + CAT model was done on CIPRES Portal 2. Non-metric multidimensional scaling (NMDS) plots as implemented in PRIMER v6 [73] for visualizing archaeal community at the different elevational scales were generated using Bray-Curtis Index.

We used a multiple regression on matrices (MRM) approach to look at the relative importance of each of the environmental factors on community similarity [74]. Before applying MRM to the dataset, we looked for redundant edaphic factors using the VARCLUS procedure [75] in the Hmisc R package. Mean annual temperature (MAT) (Spearman’s ρ^2^ = 1.00), total carbon and nitrogen (Spearman’s ρ^2^ = 0.84 & 0.85 respectively), extractable ammonium (Spearman’s ρ^2^ = 0.74) were highly correlated with elevation (Fig. S1), and thus we removed them from the MRM analysis. With the 5 environmental variables left (on the basis of VARCLUS results), we estimated an environmental distance (Euclidean distance) matrix using Primer v6 [73] and performed MRM using this environmental distance matrix and genetic matrices calculated as specified above (i.e., UniFrac and Bray-Curtis). Non-significant factors were removed sequentially and the MRM analysis was repeated until only significant factors were left in the model. Significance was tested by permutations (9999 permutations) and P-values of two-tailed tests are reported for this analysis.

Rarefaction curve, heatmap, regression analysis, VARCLUS and MRM procedures were performed using R software package 2.10.1. A neighbor-joining phylogenetic tree for inferring phylogeny for our large dataset was constructed after aligning representative phylotypes with reference sequences (J-PHYDIT software) downloaded from NCBI and EMBL in the MEGA 4 software package [76].

## Supporting Information

Figure S1 **Cluster analysis of the all 9 measured environmental variables.** The analysis was performed and plotted using VARCLUS in the Hmisc R package. Abbreviations used in the figure; NO_3_: extractable nitrate (soil), P: extractable phosphorus (soil), K: extractable potassium (soil), NH_4_: extractable ammonium (soil), altitude measured as meters above sea level, MAT: mean annual temperature, C and N are total carbon and nitrogen content of soil.
(TIF)

Table S1 **Relative abundances of archaeal phyla classified against Eztaxon-e database across all 30 soil replicates of 6 elevational points on Mt. Fuji.**
(XLS)

Table S2 **Phylotype richness (OTUs) and Diversity indices calculated for subsamples standardized for 309 reads.**
(XLS)

Document S1 **Archaeal database comparison.**
(DOC)
