# Correction: Elevational Patterns in Archaeal Diversity on Mt. Fuji

**DOI:** 10.1371/annotation/405cf97b-8e07-492d-aa4f-3ff0c2545292

**Published:** 2013-06-21

**Authors:** Dharmesh Singh, Koichi Takahashi, Jonathan M. Adams

The number of the grant indicated in Funding is incorrect. The correct grant number is: NRF-2012-0005118. 

