# Supplementary figures and images for: Elevational Patterns in Archaeal Diversity on Mt. Fuji
Source: PLoS One. 2012 Sep 6;7(9):e44494. doi: 10.1371/journal.pone.0044494 (PMC3435261; doi:10.1371/journal.pone.0044494)

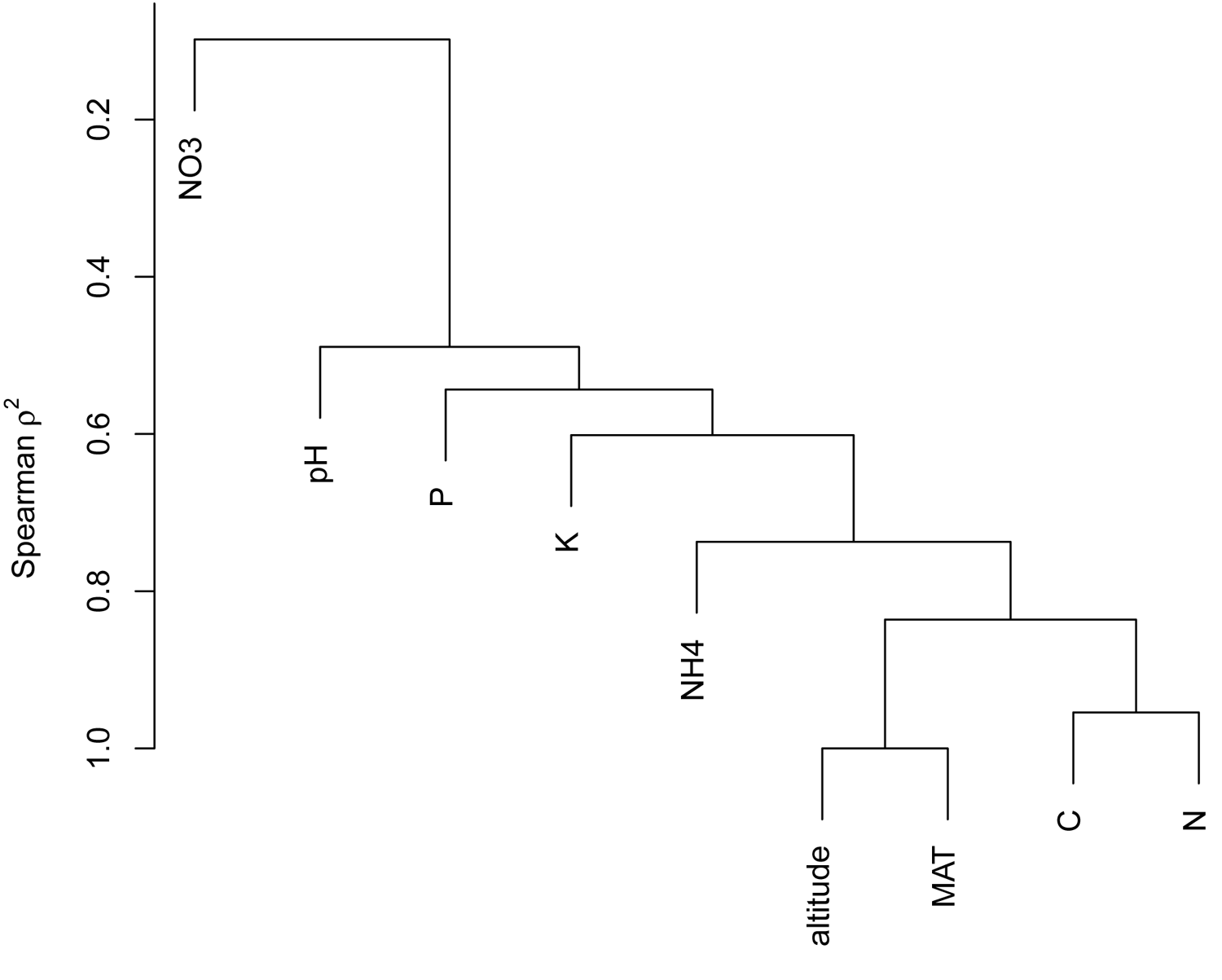

Supplement: Figure S1 — Cluster analysis of the all 9 measured environmental variables. The analysis was performed and plotted using VARCLUS in the Hmisc R package. Abbreviations used in the figure; NO3: extractable nitrate (soil), P: extractable phosphorus (soil), K: extractable potassium (soil), NH4: extractable ammonium (soil), altitude measured as meters above sea level, MAT: mean annual temperature, C and N are total carbon and nitrogen content of soil. (TIF) [file pone.0044494.s001.tif]
