# Supplementary material for: Elevational Patterns in Archaeal Diversity on Mt. Fuji
Source: PLoS One. 2012 Sep 6;7(9):e44494. doi: 10.1371/journal.pone.0044494 (PMC3435261; doi:10.1371/journal.pone.0044494)
Supplement: Document S1 — Archaeal database comparison. (DOC) [file pone.0044494.s004.doc]

# Supplementary Document S1: Archaeal Database Comparison

## Introduction

An important feature that has emerged from previous studies is that soil samples are typically dominated by a few groups of Thaumarchaeota (formerly described as Crenarchaeota ). It is important to note, however, that inferences in these previous studies have typically been drawn from comparing the observed sequence diversity against two main classifiers, Ribosomal Database Project (RDP) and SILVA ARB which have been shown recently to be problematic in terms of 454-pyrosequencing reads as their taxonomy hasn’t been updated for the proposed phylum Thaumarchaeota and they both still classify thaumarchaeotal sequences as Crenarchaeota . Whilst keeping these two points in mind, we classified our sequences recovered after 454-barcoded pyrosequencing (16S rRNA gene) to using all three archaeal databases namely RDP, SILVA and EzTaxon-e.

## Methods

We followed the Costello analysis example on the Mothur platform to process and analyze the reads obtained after pyrosequencing except for the step of removing chimeric sequences. Concisely, after removing the chimeric sequences, the fasta file containing quality sequences (89672 in total) was classified against three different databases RDP , SILVA and EzTaxon-e (at a bootstrap cut off of 80%) and results compared afterwards:

i) RDP classifier (<http://rdp.cme.msu.edu/classifier/classifier.jsp>): Removed 10 more sequences (read length <250bp) from the above file before using RDP-Classifier to assign taxonomic classifications to the sequences for ecological analysis- as a shorter sequence length than 250bp is generally recommended for a lower cut off value of 50% while classifying .

ii) SILVA database: We downloaded the SILVA SEED archaeal reference files available with the Costello analysis example on the Mothur wiki-link (<http://www.mothur.org/wiki/Silva_reference_files>) and followed the example there in to classify the sequences.

iii) EzTaxon-e database: We obtained archaeal references files from the Chunlab Inc. (Seoul, South Korea; Jongsik Chun, personal communication) and followed the instructions on the Costello analysis example.

## Results

A total of 89672 quality archaeal sequences (with an average length of 444bp) were obtained from the 30 samples, with an average of 2989 sequences per soil sample and with coverage ranging from 398 to 8488 reads per sample. The automated RDP classifier and the SILVA SEED database proved problematic for the taxonomic classification of the pyrosequencing reads, as most of the sequences were incorrectly placed as unclassified archaea or Crenarchaeota phylum. However, all three classifiers agreed upon the conclusion that all of the quality sequences belonged to domain archaea (at a bootstrap cut off of 50%). While classifying a sequence, for each rank assignment up to species level, a classifier like RDP automatically estimates the classification reliability using bootstrapping. Bootstrap value gives a sense of confidence, so for eg., if a particular OTU could be classified up to Nitrososphaera with a bootstrap cutoff value set at 50%, then we can say that we are 50% confident that the classified sequence is Nitrososphaera. At a bootstrap cutoff of 80%, RDP put around 195 sequences as unclassified root [approx., 0.2%] whereas the other two databases were still binning every sequence into domain archaea. All further results refer to data at a bootstrap cut off rate of 80%. Results of the classifications using the three different databases are as follows:

i) RDP: Out of the total sequences 89657 sequences, around 91% (81751) were classified as unclassified archaea with 7 and 7711sequences classified as Crenarchaeota and Euryarchaeota respectively.

ii) SILVA: Since in this case the Mothur platform was previously used to trim, screen and align sequences against a SILVA SEED compatible alignment database before classifying against SILVA archaea reference and taxonomy files, we were left with a different total of 89016 sequences. Unlike RDP all of the sequences could be classified into archaea, with Crenarchaeota (85335 sequences, 95.8%), Euryarchaeota (3501sequences, 3.9%) and unclassified archaea (180, 0.2%).

iii) EzTaxon-e: We followed the same procedure as above, but utilized EzTaxon-e archaeal alignment, reference and taxonomy files, leaving us with the same number of sequences (89016). The most noticeable difference from the other two databases was that most of the sequences belonged to phyla Thaumarchaeota (85840sequences, 96.4%), followed by Euryarchaeota (3515sequences, 3.9%), and unclassified archaea (21sequences, 0.02%). Surprisingly, no sequences were classified as Crenarchaeota (* at a bootstrap cut off of 50% three sequences did fall into phylum Crenarchaeota).

The contrasting results of the three systems gave much the same picture as discussed by Kan et al. . Based on our results and those by Kan et al. , we used the EzTaxon-e database to classify our recovered sequences.

## References

1. Auguet JC, Barberan A, Casamayor EO (2010) Global ecological patterns in uncultured Archaea. Isme Journal 4: 182-190.

2. Bates ST, Berg-Lyons D, Caporaso JG, Walters WA, Knight R, et al. (2011) Examining the global distribution of dominant archaeal populations in soil. Isme Journal 5: 908-917.

3. Brochier-Armanet C, Boussau B, Gribaldo S, Forterre P (2008) Mesophilic crenarchaeota: proposal for a third archaeal phylum, the Thaumarchaeota. Nature Reviews Microbiology 6: 245-252.

4. Wang Q, Garrity GM, Tiedje JM, Cole JR (2007) Naive Bayesian classifier for rapid assignment of rRNA sequences into the new bacterial taxonomy. Applied and Environmental Microbiology 73: 5261-5267.

5. Pruesse E, Quast C, Knittel K, Fuchs BM, Ludwig W, et al. (2007) SILVA: a comprehensive online resource for quality checked and aligned ribosomal RNA sequence data compatible with ARB. Nucleic Acids Res 35: 7188-7196.

6. Kan JJ, Clingenpeel S, Macur RE, Inskeep WP, Lovalvo D, et al. (2011) Archaea in Yellowstone Lake. Isme Journal 5: 1784-1795.

7. Schloss PD, Westcott SL, Ryabin T, Hall JR, Hartmann M, et al. (2009) Introducing mothur: Open-Source, Platform-Independent, Community-Supported Software for Describing and Comparing Microbial Communities. Applied and Environmental Microbiology 75: 7537-7541.

8. Kim OS, Cho YJ, Lee K, Yoon SH, Kim M, et al. (2012) Introducing EzTaxon-e: a prokaryotic 16S rRNA Gene sequence database with phylotypes that represent uncultured species. International Journal of Systematic and Evolutionary Microbiology 62: 716-721.

9. Claesson MJ, O'Sullivan O, Wang Q, Nikkila J, Marchesi JR, et al. (2009) Comparative Analysis of Pyrosequencing and a Phylogenetic Microarray for Exploring Microbial Community Structures in the Human Distal Intestine. Plos One 4.

Table 1: A concise comparison of results using three different archaeal databases

| **Database** | **Phylum** | **Class** | **Total** | **Total** |
| --- | --- | --- | --- | --- |
|  |  |  |  |  |
| **SILVA** | root | root | 89016 | 89016 |
|  | Crenarchaeota | AK31 | 85335 | 2 |
|  |  | AK59 |  | 1 |
|  |  | Marine Group_1 |  | 3 |
|  |  | Soil Crenarchaeotic Group |  | 48795 |
|  |  | South African Gold Mine Gp_1 |  | 6165 |
|  |  | terrestrial group |  | 26044 |
|  |  | Crenarchaeota_uc* |  | 4326 |
|  | Euryarchaeota | Halobacteria | 3501 | 1 |
|  |  | Thermoplasmata |  | 3214 |
|  |  | Euryarchaeota_uc |  | 286 |
|  | Archaea_uc | unclassified | 180 | 180 |
|  |  |  |  |  |
| **RDP** | root | root | 89664 | 89664 |
|  | unclassified root |  | 195 | 195 |
|  | Archaea_uc |  | 81751 | 81751 |
|  | Crenarchaeota | Thermoprotei | 7 | 7 |
|  | Euryarchaeota | Methanomicrobia | 7711 | 314 |
|  |  | Euryarchaeota_uc |  | 7397 |
|  |  |  |  |  |
| **EzTaxon-e** | root | root | 89016 | 89016 |
|  | Euryarchaeota | Euryarchaeota_uc | 3515 | 297 |
|  |  | DHVEG_3 |  | 3 |
|  |  | DHVEG_6b |  | 1 |
|  |  | Thermoplasmata |  | 3214 |
|  | Thaumarchaeota | Thaumarchaeota_uc | 85480 | 34 |
|  |  | FFSB_c |  | 29470 |
|  |  | Marine GroupI.1a |  | 6211 |
|  |  | Soil GroupI.1b |  | 49765 |
|  | Archaea_uc | unclassified | 21 | 21 |
|  |  |  |  |  |

*_uc stands for unclassified

Figure 1: Doughnut chart used to compare 3 different archaeal databases using our dataset form Mt. Fuji.
